# Noradrenaline infusion prevents anesthesia-induced hypotension in severe aortic stenosis patients undergoing transcatheter aortic valve replacement: a retrospective observational study

**DOI:** 10.1186/s40981-024-00721-4

**Published:** 2024-06-13

**Authors:** Kenta Onishi, Masashi Yoshida, Hisakatsu Ito, Masaaki Kawakami, Tomonori Takazawa

**Affiliations:** Department of Anesthesiology, University of Toyama, Toyama, Japan

**Keywords:** Noradrenaline, Aortic valve stenosis, Induction, General anesthesia, Hypotension

## Abstract

**Background:**

Patients with severe aortic valve stenosis (AS) are particularly prone to developing hypotension during general anesthesia induction, which increases postoperative morbidity and mortality. Although the preventive effect of a single vasopressor dose on anesthesia-induced hypotension has been reported, the effects of continuous vasopressor infusion are unknown. This study aimed to assess the effect of noradrenaline (NAd) infusion on hemodynamic stability during general anesthesia induction in severe AS patients undergoing transcatheter aortic valve replacement (TAVR).

**Methods:**

This single-center, retrospective study included severe AS patients who underwent elective TAVR. Patients in the NAd group received a continuous prophylactic NAd infusion of 0.1 μg/kg/min from the time of anesthesia induction. The control group received inotropes and vasopressors as indicated by the occurrence of hypotension. The primary outcome was the lowest mean blood pressure (MBP) before the start of surgery.

**Results:**

The study included 68 patients in the NAd group and 113 in the control group. The lowest MBP before the start of surgery was significantly higher in the NAd group than in the control group (63 ± 15 vs 47 ± 13 mmHg, *P* < 0.01). MBP immediately before intubation was also significantly higher in the NAd group (75 ± 17 vs 57 ± 16 mmHg, *P* < 0.01). Differences in postoperative complications between the groups were negligible.

**Conclusion:**

Continuous administration of NAd at 0.1 μg/kg/min in patients with severe AS might prevent hypotension during general anesthesia induction for TAVR.

**Supplementary Information:**

The online version contains supplementary material available at 10.1186/s40981-024-00721-4.

## Background

The most common intraoperative event in aortic valve stenosis (AS) patients is hypotension [1–3], particularly during anesthesia induction [3–5]. Left ventricular filling with adequate preload and cardiac systole is necessary to overcome valve resistance and maintain adequate hemodynamics in patients with AS [6]. Hypotension during general anesthesia, for even a short time, can cause myocardial damage and acute kidney injury [7] and is associated with increased 30-day postoperative mortality [8].

The induction of general anesthesia in AS patients results in hypotension due to reduced vascular resistance and preload [9]. Since noradrenaline (NAd) helps maintain vascular resistance and cardiac output in reduced preload situations [10], it is a potential therapeutic agent for maintaining adequate hemodynamics during general anesthesia in AS patients. Although the usefulness of a single dose of vasopressors during general anesthesia in patients with AS has been reported [11], no study has examined the effects of continuous NAd administration during general anesthesia induction in patients with AS.

We hypothesized that continuous prophylactic NAd administration during anesthesia induction might prevent post-induction hypotension in patients with severe AS, and retrospectively investigated severe AS patients who received general anesthesia for transcatheter aortic valve replacement (TAVR) to confirm our hypothesis.

## Methods

### Study design and ethics approval

This retrospective observational study included patients who underwent TAVR at Toyama University Hospital between April 2015 and March 2020. Patients receiving a continuous NAd infusion of 0.1 μg/kg/min from the time of anesthesia induction until at least the start of the surgical procedure were defined as the NAd group. All other patients were included in the control group. The Review Board of the Center for Clinical Research of Toyama University Hospital approved this study (approval no.: R2020196).

### Subjects

The inclusion criteria were patients with AS who underwent TAVR. TAVR was performed for all patients with severe AS who had an aortic valve area of < 1.0 cm^2^; Vmax > 4.0 m/sec; mean aortic valve pressure gradient > 40 mmHg, or low-gradient; and were older than 80 years and were too sick to undergo open heart surgery. Thus, the American Society of Anesthesiologists physical status (ASA-PS) was four or higher in all cases. The exclusion criteria were as follows: TAVR under only sedation and local anesthesia, anaphylaxis, insufficient data, non-matching with the study design, circulatory failure due to procedures such as aortic root rupture, cardiac tamponade, use of an assisted circulation device, ventricular perforation, and emergency thoracotomy. The final decisions regarding the performance of TAVR were determined by a cardiac team comprising cardiologists, cardiac surgeons, anesthesiologists, nurses, and clinical engineers.

### Anesthesia management and data acquisition

All procedures were performed under sterile conditions in a hybrid operating room that combined an operating room with an angiography unit. Peripheral venous and arterial pressure lines were secured before anesthesia induction. Propofol 1 mg/kg, rocuronium 0.6–1.0 mg/kg, and remifentanil 0.3–0.4 μg/kg/min were used for rapid sequence induction. General anesthesia was maintained with inhalation anesthetics, either sevoflurane or desflurane, and remifentanil 0.2 μg/kg/min. We controlled the end-tidal concentrations of volatile anesthetics at 0.5 to 0.6 times their minimum alveolar concentration in each patient.

The blood pressure of patients in the NAd group was controlled as follows: We administered NAd at 0.1 µg/kg/min from the induction of general anesthesia until at least the start of surgery to prevent hypotension. Many anesthesiologists at our hospital use this dose of NAd because it is considered the optimal concentration based on our experience. The NAd dose was increased, as required, in case of hypotension. Other aspects of anesthesia management, including the use of vasopressors such as phenylephrine and ephedrine, were at the discretion of the anesthesiologist in charge. In the control group, circulatory inotropes and vasopressors, including NAd, were administered after anesthesia induction, as indicated based on the occurrence of hypotension.

The patients’ medical and anesthesia charts were retrospectively reviewed to extract data on their clinical characteristics, preoperative echocardiography, cardiac catheterization findings, preoperative comorbidities, intraoperative hemodynamics, perioperative blood chemistry analyses, perioperative processes, and other complications. Cardiac index measurements were performed preoperatively using Swan-Ganz catheters in all cases.

### Outcomes

The primary outcome was the lowest MBP before the start of surgery. The secondary outcomes included hypotension, the use of additional hemodynamic agents, and postoperative complications. Hypotension was defined as an MBP of < 65 mmHg [7]. We calculated the total number of minutes for which the MBP was < 65 mmHg before the start of surgery. Postoperative complications included myocardial infarction, acute kidney injury (AKI), cerebral infarction, hemodialysis, and 30-day mortality. Myocardial infarction was diagnosed by the presence of a peak troponin *T* value exceeding 15 times the normal upper limit, along with new pathological Q waves on ECG and symptoms suggestive of myocardial infarction [12]. AKI was diagnosed by comparing preoperative and postoperative serum creatinine values. The most recent value of creatinine concentration measured before surgery was considered the preoperative creatinine value. The postoperative value was defined as the highest creatinine concentration measured within seven days after surgery. Patients were considered to have AKI when the highest postoperative value was > 1.5-fold or 0.3 mg/dL greater than the preoperative value [7, 13]. Cerebral infarction was diagnosed based on clinical symptoms and the results of computed tomography or magnetic resonance imaging performed before discharge. Arterial blood gas analysis, including lactate levels, was performed before the start of surgery in all cases.

### Statistical analysis

We performed a sensitivity analysis to test whether using another definition of hypotension, rather than an absolute MBP value of < 65 mmHg, would change our conclusions. Two definitions of hypotension were employed in the sensitivity analysis: 20% and 30% decreases from baseline MBP. Fisher’s exact test, unpaired *t*-test, Mann–Whitney *U* test, and two-way analysis of variance with the Bonferroni post-hoc test were used for statistical analysis according to the data type. An *F*-test was performed beforehand, and a nonparametric test was used to evaluate whether the null hypothesis, which proposes that the standard deviations of the two groups were equal when following a normal distribution, should be rejected; otherwise, a parametric test was used. We used G*power 3 (Münster University, Germany) for the power analysis, and Graph Pad Prism 9.1.0 (GraphPad Software Inc., CA, USA) for all other analyses. Data are presented as the mean ± standard deviation or median (maximum, minimum). Statistical significance was set at *P* < 0.05.

To estimate the number of patients needed for the study, we first conducted a pilot study with six patients in the NAd group and ten patients in the control group. The lowest mean blood pressure (MBP) before the start of surgery in the NAd and control groups were 64 ± 19 vs 55 ± 19 mmHg, respectively. Based on the difference in MBP between the two groups and the standard deviation of the MBP, we estimated an effect size of 0.44. The evaluation showed that the number of patients required in the NAd group was 68, with power analysis using an effect size of 0.44, α = 0.05 and β = 0.8, and an allocation ratio (NAd group/control group) of 0.65.

## Results

**Fig. 1:**
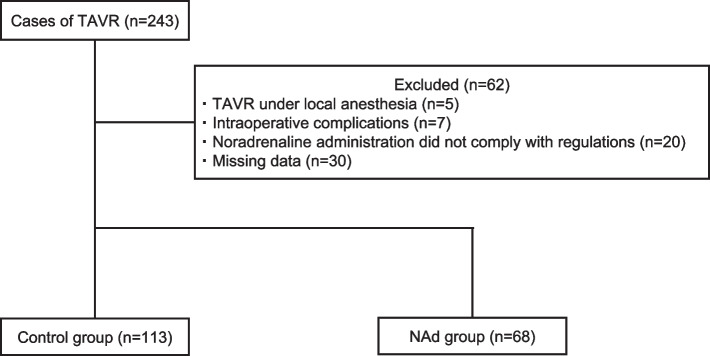
Flow diagram of this study. Seven patients in this study developed intraoperative complications, including ventricular perforation (*n* = 2), emergency thoracotomy (*n* = 2), anaphylaxis (*n* = 2), cardiac base rupture (*n* = 1), cardiac tamponade (*n* = 1), and use of assisted circulation devices (*n* = 1), with some cases developing multiple complications. Abbreviations: TAVR, transcatheter aortic valve replacement; NAd, noradrenaline

The study included 113 and 68 patients in the control and NAd groups, respectively (Fig. 1), after excluding 62 patients for the following reasons: five underwent TAVR under local anesthesia, seven had intraoperative complications (cardiac base rupture, cardiac tamponade, use of assisted circulation, ventricular perforation, emergency thoracotomy, and anaphylaxis), 20 patients in whom the dose of NAd was not 0.1 μg/kg/min, and 30 patients with missing data.

Baseline patient characteristics are presented in Table 1. Histories of cerebral infarction and atrial fibrillation were significantly more frequent in the NAd group than in the control group. The aortic valve opening area was smaller in the NAd group (0.5 [0.8, 0.3] vs 0.6 [1.3, 0.2] cm^2^, *P* = 0.03). Left ventricular ejection fraction was preserved (> 50%) in most patients in both groups. The cardiac index, evaluated using the Swan-Ganz catheter, was similar in both groups. E/e’ (ratio of mitral inflow *E* velocity to Doppler tissue imaging-derived mitral annular wave) was > 8 in all patients, except for one patient in the control group.

**Table 1:** Patients’ baseline characteristics

	Control (***n*** = 113)	NAd (***n*** = 68)	***p*** value
Characteristics			
Age (years)	85.5 (4.7)	85.9 (4.4)	0.56
Female, ***n*** (%)	88 (77.9%)	46 (67.7%)	0.16
BSA (m^2^)	1.4 (0.2)	1.4 (0.2)	0.60
6MWD (m)	215.7 (86.7)	217.5 (81.5)	0.90
NYHA class (III/IV), ***n*** (%)	39 (34.5%)	32 (47.1%)	0.12
CSHA-CFA (> 5), ***n*** (%)	23 (20.4)	7 (10.3)	0.10
STS mortality score	6.272 (3.0)	6.473 (3.7)	0.71
Preoperative comorbidities			
COPD, ***n*** (%)	25 (22.1%)	17 (25.0%)	0.72
Ischemic heart disease, ***n*** (%)	46 (40.7%)	38 (55.9%)	0.07
Cerebral infarction, ***n*** (%)	16 (14.2%)	19 (27.9%)	0.03
Diabetes mellitus, ***n*** (%)	26 (23.0%)	14 (20.6%)	0.85
ASO, ***n*** (%)	3 (2.7%)	2 (2.9%)	> 0.99
Atrial fibrillation, ***n*** (%)	20 (17.7%)	21 (30.9%)	0.05
S Cre (mg/dL)	0.8 [2.0, 0.5]	0.9 [4.0, 0.5]	0.27
BNP (pg/mL)	274 [2614, 7]	429 [5005, 19]	0.08
Echocardiography			
LVDd (mm)	46 (7)	48 (6)	0.06
LVDs (mm)	30 (7)	31 (7)	0.20
EF (%)	63 (11)	62 (13)	0.58
LV asynergy, ***n*** (%)	20 (17.7%)	19 (27.9%)	0.14
IVS thickness (mm)	12 (1)	11 (1)	0.20
PW thickness (mm)	11 (1)	11 (1)	0.48
Sigmoid septum, ***n*** (%)	10 (8.1%)	3 (4.4%)	0.39
Mean E/e’	16.7 [47.9, 7.3]	16.8 [36.6, 17.0]	0.56
TR velocity (m/s)	258 [346, 203]	248 [360, 100]	0.55
LAVI (mL/m^2^)	29.4 [225.0, 18.8]	32.2 [52.1, 19.3]	0.03
AVA (cm^2^)	0.6 [1.3, 0.2]	0.5 [0.8, 0.3]	0.03
Mean PG of the aortic valve (mmHg)	49 (16)	51 (16)	0.48
AR (moderate/severe),*** n*** (%)	8 (7.1%)	3 (4.4%)	0.54
MR (moderate/severe), ***n*** (%)	8 (7.1%)	5 (7.4%)	> 0.99
TR (moderate/severe), ***n*** (%)	1 (0.9%)	3 (4.4%)	0.15
Swan-Ganz catheter			
Cardiac index (L/min)	2.7 (0.5)	2.6 (0.6)	0.39

Values are presented as *n* (%), mean (standard deviation), or median [max, min]

*Abbreviations*: *BSA* Body surface area, *6MWD* 6-min walk distance, *NYHA* New York Heart Association, *CSHA* Canadian Study for Health and Aging, *CFA* Clinical frailty score, *STS* Society of Thoracic Surgeons, *COPD* Chronic obstructive pulmonary disease, *ASO* Arteriosclerosis obliterans, *S Cre* Serum creatinine, *BNP* Brain natriuretic peptide, *LVDd* Left ventricular diameter in diastole, *LVDs* Left ventricular diameter in systole, *EF* Ejection fraction, *LV* Left ventricle, *IVS* Interventricular septum, *PW* Posterior wall, *E/e’* Early transmitral velocity to tissue Doppler-estimated mitral annular early diastolic velocity ratio, *TR* Tricuspid regurgitation, *LAVI* Left atrial volume index, *AVA* Aortic valve area, *PG* Pressure gradient, *AR* Aortic regurgitation, *MR* Mitral regurgitation, *PW* Posterior wall

**Fig. 2:**
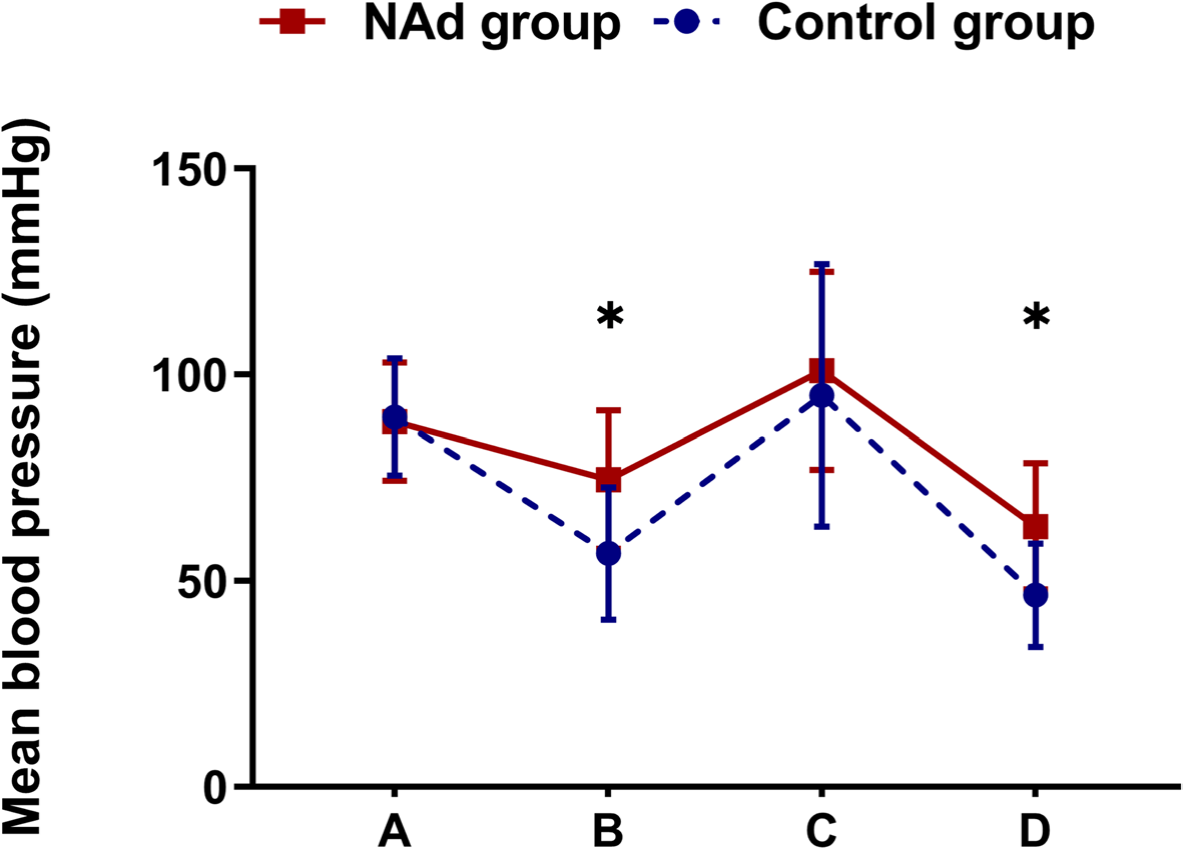
Changes in mean blood pressure during general anesthesia. The solid squares and circles indicate mean blood pressure (MBP) in NAd and control group patients, respectively, at the following time points: **A** at admission, **B** immediately before intubation, **C** immediately after intubation, and **D** the lowest value before the start of surgery. The error bars show the standard deviation, and asterisks indicate significant differences in MBP between groups at the same time point. There was a significant difference in MBP for all time point combinations in the NAd group and for all time point combinations except **A** and **C** in the control group. However, these results are not displayed. *P < 0.01, two-way ANOVA with a post-hoc Bonferroni multiple comparison test. Abbreviations: ANOVA, analysis of variance

MBP trends are shown in Fig. 2. MBP immediately before intubation (75 ± 17 vs 57 ± 16 mmHg in the NAd and control groups, respectively, *P* < 0.01, at point B) and the lowest MBP before the start of surgery (63 ± 15 vs 47 ± 13 mmHg, respectively, *P* < 0.01, at point D) were significantly higher in the NAd group. MBP values at other measurement points (at admission and immediately after intubation) did not differ between the two groups.

The percentage of patients who experienced post-induction hypotension was lower in the NAd group (75.0% vs 99.1%, *P* < 0.01) (Table 2). The duration of hypotension before surgery was significantly shorter in the NAd group (11 ± 11 vs 29 ± 12 min,* P* < 0.01). The dose of NAd between induction and the beginning of surgery was higher in the NAd group (0.38 [0.20, 0.74] vs 0.04 [0.01, 0.26], respectively, *P* < 0.01). Phenylephrine and ephedrine doses administered before the surgery did not, however, differ between the two groups. Fluid volume, urine volume, and total volume balance did not differ between the two groups.

**Table 2:** Intraoperative hypotension and treatment

	Control (***n*** = 113)	NAd (***n*** = 68)	***p*** value
Number of patients who experienced post-induction hypotension, ***n*** (%)	112 (99.1%)	51 (75.0%)	< 0.01
Duration of hypotension before the start of surgery (min)	28 (12)	11 (11)	< 0.01
Number of patients receiving hemodynamic agents before the start of surgery			
Phenylephrine, ***n*** (%)	90 (79.6%)	62 (91.1%)	0.06
Ephedrine, ***n*** (%)	85 (75.2%)	49 (72.0%)	0.73
Noradrenaline, ***n*** (%)	113 (100%)	68 (100%)	> 0.99
Dosage of hemodynamic agents before the start of surgery			
Phenylephrine (mg)	0.06 (0.13)	0.08 (0.13)	0.42
Ephedrine (mg)	3.3 (4.8)	2.2 (4.1)	0.10
Noradrenaline (mg)	0.04 [0.01, 0.26]	0.38 [0.20, 0.74]	< 0.01
Intraoperative volume data			
Infusion volume (mL)	1911 (401)	1892 (333)	0.74
Urine volume (mL)	570 [1900, 50]	710 [2300, 10]	0.14
Blood transfusion (mL)	75 (177)	92 (166)	0.53
Total fluid balance (mL)	1113 (617)	1164 (574)	0.59

Values are presented as the *n* (%), mean (standard deviation) or median [max, min]

Table 3 shows the postoperative complications in the patients. No myocardial infarction or need for hemodialysis induction was seen in either group, although AKI occurred in 10 (8.9%) and two (2.9%) patients in the control and NAd groups, respectively. Cerebral infarction occurred in 10 (8.9%) patients in the control group and two (2.9%) in the NAd group. Patients who developed cerebral infarction showed no intraoperative reduction in regional cerebral oxygen saturation on near-infrared spectroscopy. All patients had asymptomatic cerebral micro-embolisms on computed tomography (CT) or magnetic resonance imaging. Death within 30 days, due to cerebral hemorrhage, occurred in only one patient in the NAd group.

**Table 3:** Postoperative complications and results of blood tests

	Control (***n*** = 113)	NAd (***n*** = 68)	***p*** value
Peak CK (IU/L)	122 [927, 18]	92 [490, 35]	0.13
Myocardial infarction, ***n*** (%)	0 (0.0%)	0 (0.0%)	1.00
Troponin T (ng/mL)	0.13 [2.4, 0.02]	0.15 [0.91, 0.02]	0.28
S Cre (mg/dL)	0.92 [2.62, 0.43]	0.95 [3.63, 0.39]	0.50
Acute kidney injury, ***n*** (%)	10 (8.6%)	2 (2.9%)	0.22
Cerebral infarction, ***n*** (%)	10 (8.9%)	2 (2.9%)	0.22
Hemodialysis, ***n*** (%)	0 (0.0%)	0 (0.0%)	1.00

Values are presented as the *n* (%) or median [max, min]

*Abbreviations*: *CK* Creatine kinase, *S Cre* Serum creatinine

## Discussion

We investigated the hypothesis that continuous administration of NAd during anesthesia induction might prevent post-induction hypotension in patients with severe AS. Our results suggest that continuous administration of NAd during induction has the potential to prevent anesthesia-induced hypotension in patients with severe AS. To the best of our knowledge, this is the first study to examine the preventive effects of continuous NAd administration on hypotension during general anesthesia induction in patients with severe AS undergoing TAVR.

In this study, there were no restrictions on the use of vasopressors in either group, except for the prerequisite of continuous NAd administration during anesthesia induction in the NAd group, and there were no significant differences in phenylephrine or ephedrine use before the start of surgery between the two groups. Despite this, however, MBP immediately before intubation and the lowest MBP before the start of surgery were significantly higher, and the duration of hypotension before the start of surgery was significantly shorter in the NAd group. These results suggest that a continuous prophylactic vasopressor infusion might be superior to a single vasopressor dose for maintaining MBP during anesthesia induction in severe AS patients. In these patients, hypotension results in a negative hemodynamic spiral, with decreased coronary blood flow and cardiac systole, and increased cardiac output [6]. We believe that prophylactic continuous administration of NAd, rather than a single dose of a vasopressor after the diagnosis of hypotension, might help avoid the negative spiral of circulatory collapse caused by hypotension in severe AS patients.

With respect to myocardial and renal damage secondary to hypotension, although MBP thresholds for hypotension have included an absolute value of 65 mmHg or a decrease of more than 20% from baseline, absolute thresholds are reportedly more strongly associated with myocardial and renal damage than relative MBP reduction [7]. Thus, we employed the absolute MBP value of 65 mmHg as the criterion to define hypotension in this study. Sensitivity analysis performed using relative MBP reduction to define hypotension indicated similar results as using the absolute MBP threshold in terms of the number of patients who developed hypotension after the induction of general anesthesia (Supplementary Table S1, Supplementary Figure S1). These results support the findings of the current study.

The results of this study suggested that a continuous NAd infusion during general anesthesia induction does not affect the incidence of postoperative complications. Although we were concerned about hypertension due to NAd administration before the study, MBP did not differ between the two groups immediately after intubation. These results suggest that an NAd dosage of 0.1 μg/kg/min does not cause severe hypertension. We were also concerned about the excessive vasoconstrictive effect of noradrenaline on renal blood flow and the potential for renal dysfunction. The incidence of AKI after TAVR is reportedly 8.3–58% [14–20]. In this study, the incidence of AKI in the NAd group was 2.9% (Table 3), which was lower, but not significantly different, from that in the control group.

In general, the surgical technique strongly influences the incidence of postoperative complications and could have biased our results. For example, arrhythmia and circulatory failure secondary to wire manipulation and rapid pacing are likely to occur during TAVR [21]. In the future, the effects of continuous NAd infusion during general anesthesia induction on circulatory failure and postoperative complications in severe AS patients should also be investigated in those undergoing non-cardiac surgery.

This study has several limitations. First, it was a retrospective observational study performed at a single center. Future prospective studies are needed to determine the effects of continuous NAd administration during induction of general anesthesia in severe AS patients. Second, the optimal dose of NAd has not been determined. Our NAd dose prevented hypotension after induction of anesthesia without increasing complications, but whether this dose is optimal remains to be verified. Third, several patient background characteristics differed significantly between the groups. Indeed, the left atrial volume index (LAVI) was greater, and the aortic valve area (AVA) was smaller in the NAd group than in the control group (Table 1). These facts suggest that the AS might have been slightly more severe in the NAd group. However, considering that the NAd group was less prone to hypotension, this supports the efficacy of prophylactic continuous NAd administration during general anesthesia induction in severe AS patients undergoing TAVR surgery.

## Conclusion

Continuous administration of 0.1 μg/kg/min NAd at the time of anesthesia induction in patients with severe AS undergoing TAVR surgery might prevent hypotension during general anesthesia induction.

### Supplementary Information

Supplementary Material 1: Supplementary Table S1. Number of patients who experienced post-induction hypotension. Supplementary Figure S1. Changes in the relative value of mean blood pressure during general anesthesia.

## Data Availability

The datasets used and/or analyzed during the current study are available from the corresponding author on reasonable request.
