# Supplementary material for: Noradrenaline infusion prevents anesthesia-induced hypotension in severe aortic stenosis patients undergoing transcatheter aortic valve replacement: a retrospective observational study
Source: JA Clin Rep. 2024 Jun 13;10:39. doi: 10.1186/s40981-024-00721-4 (PMC11176125; doi:10.1186/s40981-024-00721-4)
Supplement: Supplementary file 1 — Supplementary Material 1: Supplementary Table S1. Number of patients who experienced post-induction hypotension. Supplementary Figure S1. Changes in the relative value of mean blood pressure during general anesthesia. [file 40981_2024_721_MOESM1_ESM.docx]

Supplementary Table 1 Number of patients who experienced post-induction hypotension

| Definition of hypotension | Control (n=113) | NAd (n=68) | p Value |
| --- | --- | --- | --- |
| Absolute MBP threshold (<65 mmHg) | 112 (99.1%) | 51 (75.0%) | 1.84 X 10^-7^ |
| Relative MBP reduction of 20% | 107 (94.7%) | 49 (72.1%) | 3.34 X 10^-5^ |
| Relative MBP reduction of 30% | 99 (87.6%) | 32 (47.1%) | 5.96 X 10^-9^ |

Values are presented as the n (%). We used Fisher’s exact test for statistical analysis.

Supplementary Figure 1 Changes in the relative value of mean blood pressure during general anesthesia

**
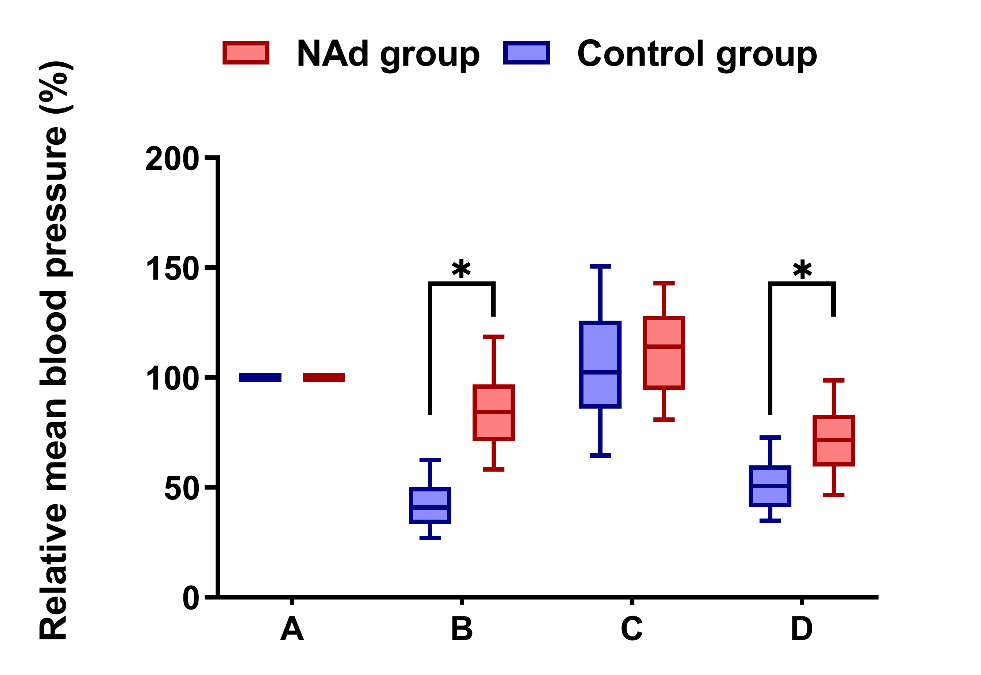
**

Relative mean blood pressure (MBP) of patients in the NAd group (red) and control group (blue) at the following time points were compared: (A) at admission, (B) immediately before intubation, (C) immediately after intubation, and (D) the lowest value before the start of surgery. Relative MBP was calculated by dividing the MBP at each time point by the MBP at baseline (time point A). The box shows the 25th percentile, median, and 75th percentile. Error bars above and below the box indicate the 90th and 10th percentiles, respectively. Asterisks indicate significant differences in relative MBP between groups at the same time point. There was a significant difference in relative MBP for all time point combinations in the NAd group, and for all time point combinations except A and C in the control group. However, the results are not displayed. *P < 0.01, Multiple Mann-Whitney U test.
